# Enhancing Paddy Rice Preservation in Small-Scale Barns: Comparative Analysis of Hot Air-Drying Techniques and Ventilation Impact on Quality and Energy Efficiency

**DOI:** 10.3390/foods13050672

**Published:** 2024-02-23

**Authors:** Wasan Duangkhamchan, Khanhatai Huangsaeng, Naoshi Kondo, Donludee Jaisut

**Affiliations:** 1 Research Unit of Process Design and Automation, Faculty of Engineering, Mahasarakham University, Maha Sarakham 44150, Thailand; wasan.d@msu.ac.th; 2 Research Unit of Smart Process Design and Automation, Mahasarakham University, Maha Sarakham 44150, Thailand; 3 Chanthaburi Provincial Agricultural Extension Office, Chanthaburi 22000, Thailand; khanhatai25kasetchan@gmail.com; 4 Division of Environmental Science and Technology, Graduate School of Agriculture, Kyoto University, Kyoto 606-8502, Japan; kondo.naoshi.6w@kyoto-u.ac.jp; 5 Department of Farm Mechanics, Faculty of Agriculture, Kasetsart University, Bangkok 10900, Thailand

**Keywords:** paddy rice storage, hot air-drying, ambient air ventilation, grain quality preservation, small-scale agriculture

## Abstract

This investigation explores the effectiveness of hot air-drying and ambient ventilation techniques in enhancing the storage quality of Khao Dok Mali 105 paddy rice within small-scale barns in Northeast Thailand. Through comprehensive analysis of moisture and temperature dynamics, the research revealed that an optimized main air pipe system significantly reduces moisture content from 25% db to a desirable 16% db, outperforming alternative systems. Spatial assessments within the barn highlighted the importance of placement, showing that front sections achieved lower moisture levels. This underscores the need for uniform moisture distribution and temperature management to prevent quality degradation. Notably, after 84 h of drying, variations in moisture content across different barn locations emphasized the critical role of environmental control. These insights pave the way for advancing grain storage practices, focusing on strategic ventilation and environmental monitoring to ensure rice quality over time. This study not only challenges traditional methods but also offers significant practical implications for optimizing small-scale rice storage, providing a pathway towards sustainable post-harvest processing in resource-constrained environments.

## 1. Introduction

Paddy rice plays an essential role in global dietary needs, and it undergoes a sequence of post-harvest processes among which drying emerges as the most crucial. Effective drying not only ensures that the rice retains its original quality but also addresses the widespread issue of losses after harvesting in many agriculture-driven regions. This drying process has broader effects, impacting both the financial outcomes at the farming level and larger trade patterns, as emphasized by research works from Sricharoen [1] and Ziegler et al. [2].

The traditional open-air sun drying method, widely used in various farming contexts, leverages existing environmental factors to systematically decrease moisture in paddy. The fascinating interplay between outdoor weather elements and the preservation of grains within is a subject of deep interest. As highlighted by researchers such as Razavizadeh et al. [3] and Scariot et al. [4], the effectiveness of these drying methods is pivotal in determining essential grain quality indicators. Notably, attributes like head rice yield and the visual appeal of whiteness, which often shape consumer preferences and market value, are influenced by these processes.

While a significant amount of research has been dedicated to large-scale industrial drying models, highlighted by studies from Muller et al. [5] and Moraes et al. [6], small-scale traditional barn drying has received comparatively less academic attention. Notable contributions by Jittanit and Angkaew [7] have examined superheated-steam drying’s impact on rice quality, presenting innovative solutions to chalkiness and yield challenges. Similarly, Hu et al. [8] have delved into the deterioration of rice flavor during storage, highlighting the complex interplay of aroma and taste compounds. Despite these advancements, a comprehensive analysis incorporating both the mechanical and sensory aspects of rice quality preservation in traditional settings has yet to be fully realized. This study seeks to bridge this gap by investigating the nuanced effects of hot air-drying and ambient ventilation on paddy rice, thereby offering a holistic view of quality maintenance over prolonged storage periods.

Our study aims to deepen the exploration of hot air-drying and ambient ventilation’s effects on paddy rice in traditional barns, building on foundational work such as that by Martens et al. [9]. Their pivotal research focused on the influence of drying time and intermittence on the physicochemical and morphological qualities of polished and brown rice, employing advanced techniques like near-infrared spectroscopy, X-ray diffraction, and scanning electron microscopy. Martens et al.’s findings underscored the critical balance between drying conditions and rice quality, revealing how accumulated drying time and temperature variations directly affect grain characteristics. Inspired by these insights, our research extends the dialogue by comparing distinct hot air-drying techniques and their comprehensive impact on grain quality standards, emphasizing the synergy between methodological rigor and practical application in rice preservation.

At the core of our study is a profound desire to thoroughly understand the complexities of hot air-drying methods, specifically within the confines of traditional barns. Our unwavering commitment revolves around assessing the effects on the quality aspects of paddy. Our innovative approach to ventilation not only examines its influence on the temperature dynamics of stored grain but also evaluates the persistence of grain quality and the economic considerations related to energy consumption over a six-month storage period.

## 2. Materials and Methods

### 2.1. Paddy Sample and Traditional Small-Scale Barn

In this study, we utilized freshly harvested paddy rice from the Khao Hom Dok Mali 105 variety (KHDM105), sourced from Kalasin Province in Northeast Thailand, as our research sample.

### 2.2. In-Barn Drying-Storage Analysis

**Figure 1:**
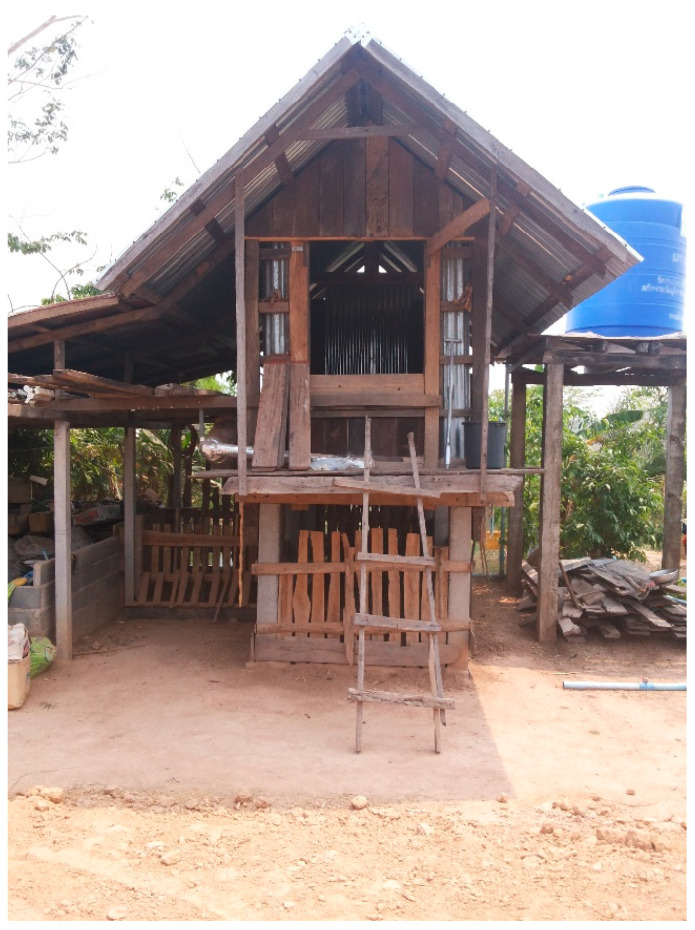
Traditional small barn.

Our investigation focused on the efficiency of a traditional in-barn drying-storage system, detailed in Figure 1, and was structured across three distinct research phases: dehydration, short-term storage without ventilation, and extended storage with occasional ventilation.

The initial phase was dedicated to freshly harvested paddy, with the aim of pinpointing the optimal forced-air arrangement. This involved a meticulous analysis of temperature and moisture distribution, employing specific criteria for uniformity and effectiveness in drying. After the drying phase, the paddy was stored in the same barn for a month without ventilation. This allowed us to observe the changes in temperature and moisture attributed to the storage conditions. In the final phase, we introduced periodic ventilation using ambient air to the stored paddy, broadening our focus to understand the long-term effects of our integrated drying and storage approaches. Specifically, we examined grain preservation, energy effectiveness, and the overall sustainability of the chosen techniques, leveraging quantitative measures to assess outcomes.

#### 2.2.1. Paddy Dehydration

**Figure 2:**
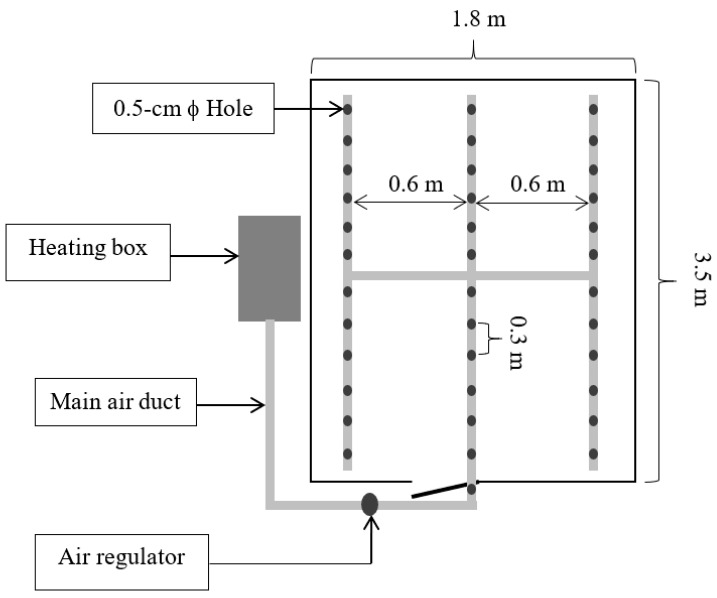
Schematic diagram of hot air supply configuration using a perforated sub pipe system.

**Figure 3:**
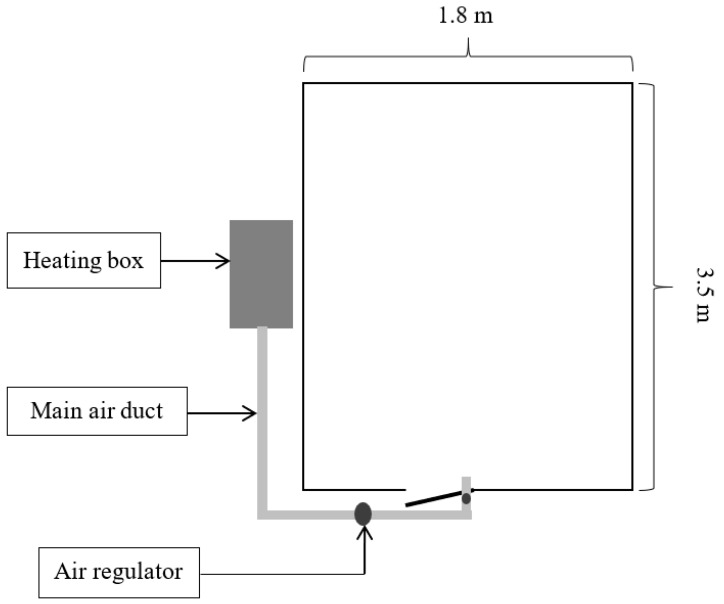
Schematic diagram of hot air supply configuration using only a main pipe system.

**Figure 4:**
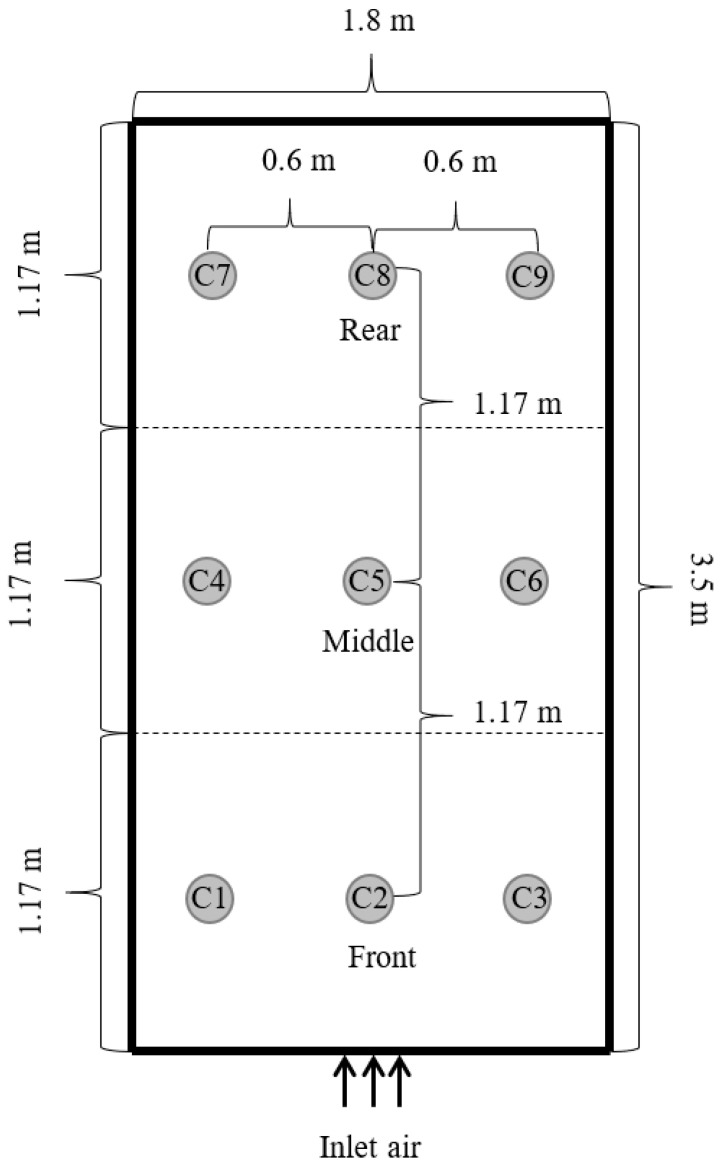
Measuring points on the horizontal plane (upper section).

**Figure 5:**
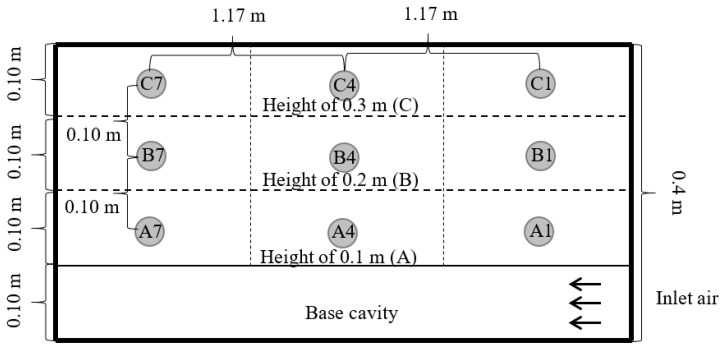
Measuring points on the vertical plane (left side).

**Figure 6:**
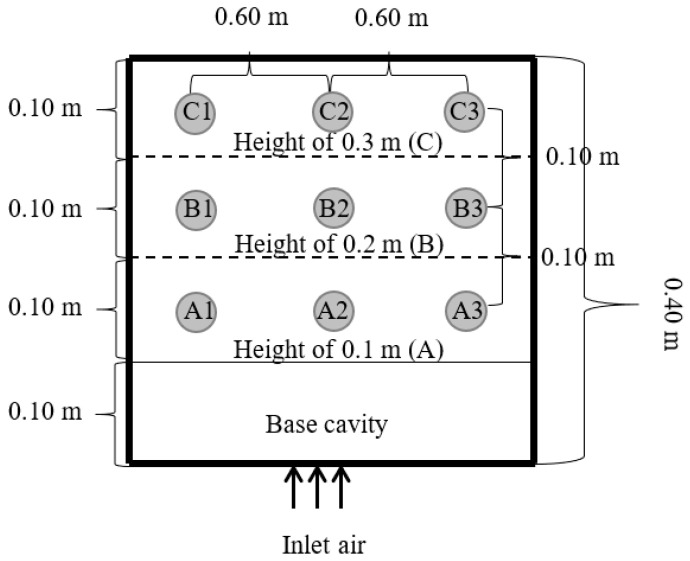
Measuring points on the vertical plane (front side).

The paddy rice (KHDM105), freshly harvested, underwent an initial moisture assessment with the Kett Grain Moisture Tester PM-600 (Kett, Tokyo, Japan), indicating a moisture content of approximately 25% on a dry basis (db). This selected sample was then weighed and placed in a traditional small-scale barn for drying. Two primary drying approaches were employed: one involved channeling hot air through a perforated sub-duct, as depicted in Figure 2, and the other directed hot air through the main duct at the barn entrance, as shown in Figure 3. The drying space, measuring 1.8 m × 3.5 m × 1.8 m, contained a grain pile about 30 cm in height. The drying process occurred between 11:00 a.m. and 5:00 p.m., a period chosen for its lower ambient relative humidity, maintaining a constant temperature of 45 °C and an airflow rate of 0.6 m^3^·min^−1^ per m^3^ of paddy. To assess drying effectiveness, moisture content and temperature were periodically measured at various points within the barn, illustrated in Figure 4, Figure 5 and Figure 6. Drying was continued until the moisture levels decreased to approximately 16% db. Upon completion, we recorded the energy consumed and collected a sample for further quality analysis. For comparative analysis, the thickness of the paddy layer was consistently maintained at approximately 5 cm during open-air sun drying.

#### 2.2.2. Short-Term Storage without Ventilation

Upon reaching the desired moisture content of around 16% on a dry basis, as recommended by guideline KHDM105 for safe storage, the paddy rice was left undisturbed. This period of observation allowed us to closely monitor the natural temperature and moisture content fluctuations within the storage environment. This decision was informed by extensive research and existing literature [10], which highlights the 16% moisture level as optimal for minimizing risks like fungal growth and ensuring the rice’s quality over time. In the first month of storage, we purposely avoided introducing external ambient air (from Week 1 to Week 4). This approach helped us focus on understanding the intrinsic behavior of the paddy pile, observing how temperature and moisture levels self-regulate without the influence of external air, thus ensuring a more controlled analysis of the rice’s storage conditions.

#### 2.2.3. Long-Term Storage with Ambient Air Ventilation

After the first month, we established an experimental protocol that involved introducing ambient air into the paddy pile once monthly at the end of each month, continuing for a total duration of six months. This procedure was achieved by only activating the fan component of the drying apparatus to channel ambient air into the stored paddy. We maintained a consistent airflow rate of 0.6 m^3^·min^−1^ per m^3^ of paddy. The ambient air ventilation sessions were strategically scheduled between 11:00 a.m. and 1:00 p.m. to take advantage of optimal external conditions and to modulate the internal temperature of the paddy pile, serving essentially as a cooling mechanism.

Data acquisition concerning temperature and moisture variations in the stored paddy was meticulously conducted at strategically chosen locations within the storage framework. Specifically, our focus was on recordings taken 20 cm below the surface of the paddy pile, averaging readings from points B1 to B9 (B layer), as illustrated in Figure 5.

### 2.3. Analysis of Quality Attributes

In this study, an in-depth analysis was conducted on the quality of rice subjected to various dehydration techniques within the paddy barn. Emphasis was placed on evaluating the percentage of head rice yield (%HRY) and the whiteness of the processed rice, compared with those obtained from rice dried via open-air sun drying, which was considered as a reference drying method. Additionally, eating properties and sensory quality assessments were conducted for the milled rice. The selection of moisture content, whiteness, and %HRY as the primary quality attributes for evaluation is grounded in their critical importance for consumer acceptance and their prevalent use by milling factories for the initial assessment of paddy rice after six months of storage. These parameters not only reflect the immediate visual and physical quality of rice that influences consumer purchasing decisions but also serve as standard benchmarks in the industry for evaluating the effectiveness of drying and storage techniques.

#### 2.3.1. Head Rice Yield

The determination of head rice yield (%HRY) was conducted through the initial separation of blighted or incomplete kernels using a dry rice separator. This was followed by the dehulling and polishing of the rice, utilizing equipment provided by NGEK SENG HUAT Limited Partnership, based in Bangkok, Thailand. For the purposes of this analysis, ‘head rice’ was defined as whole kernels plus broken kernels that are at least 75–80% of the size of a whole kernel, aligning with common industry standards [7,11]. A 250 g subset of cleaned rice was subsequently processed through dehulling and polishing. After the rice reached ambient temperature, the grains were sorted by size to separate head rice from broken grains. The weight of the head rice was then recorded using a 3-digit balance (Model DNA503, XingYun, Jiangsu, China), enabling the calculation of %HRY based on the mass of whole and qualifying broken grains, as detailed in Equation (1).

(1) $$\%\mathrm{HRY}=\frac{\mathrm{Weight}\;\mathrm{of}\;\mathrm{head}\;\mathrm{rice}}{\mathrm{Total}\;\mathrm{weight}\;\mathrm{of}\;\mathrm{paddy}}\times 100$$

#### 2.3.2. Whiteness Index

The whiteness index (WI) was employed to evaluate the whiteness level of milled rice, as calculated in Equation (2) [11]. The color of the milled rice was determined within the CIE (L*, a*, b*) color system using a chromameter (CR-400, Konica Minolta Sensing, Osaka, Japan).

(2) $$\mathrm{WI}=100-\sqrt{\left[{\left(100-L*\right)}^{2}+{\left(a*\right)}^{2}+{\left(b*\right)}^{2}\right]}$$

#### 2.3.3. Cooking and Eating Properties

The apparent amylose content of rice starch was analyzed using the iodine reagent approach, following [12]. Specifically, 25 mg of rice flour was gelatinized overnight in 2 mL of 1.0 N NaOH at 50 °C. After boiling for 10 min, it was cooled to room temperature and underwent lipid extraction thrice with a butanol–petroleum ether mix (1:3 ratio). Following the addition of 1.5 mL of 0.4 N KI, the apparent amylose content was determined using a titration method with 1.57 mM KIO_3_. The endpoint was auto-detected and the KIO_3_ volume was then equated to amylose content. Reference solutions were prepared using pure amylose and amylopectin [13].

#### 2.3.4. Sensorial Evaluation

A group of 12 panelists (comprising twelve psychologically and physically healthy adults: four males and eight females) from our research group at Kasetsart University, Thailand, who were proficiently trained in rice sensory evaluation, participated in this study. Cooked rice, prepared with a rice-to-water weight ratio of 1:1.6, was evaluated using the 9-point hedonic scale. Panelists assessed the cooked rice for various attributes such as fragrance, whiteness, glossiness, cohesiveness, and tenderness. The ratings were assigned on a structured 9-point scale, where 1 indicated a very low intensity and 9 represented a very high intensity. The panel first evaluated the rice’s external features, considering its whiteness and glossiness. Cohesiveness was assessed by observing how separated the grains were in three different servings. The rice’s fragrance was judged by smelling it before tasting, and its tenderness was assessed by noting the effort required to bite the rice sample between the molars during the initial bites.

### 2.4. Analysis of Electric Energy Consumption during In-Barn Paddy Dehydration Process

The following equations and their description were used to evaluate the electric energy consumption during in-barn drying and storage.

#### 2.4.1. Drying Rate

Drying rate (DR) was calculated from the amount of water evaporated over a specific drying duration (t) and expressed in terms of kg·h^−1^, as expressed in Equation (3), where m_p_ with subscript i and f represent initial and final paddy mass (kg), respectively.

(3) $$\mathrm{DR}\;=\frac{m_{pi}-m_{pf}}{t}$$

#### 2.4.2. Specific Primary Energy Consumption

The specific primary energy consumption (SPEC) refers to the amount of energy consumed (E) in kWh per kg of water evaporated (M_evap_). Therefore, it was expressed in MJ·kg^−1^, as follows:

(4) $$\mathrm{SPEC}=\frac{3.6E}{M_{\mathrm{evap}}}$$

#### 2.4.3. Electricity Cost for Drying

The drying expenses (Exp_d_) in this study were derived solely from electrical costs, as electricity is the exclusive energy source utilized. The cost was measured in Baht·ton^−1^·kg^−1^, based on an electricity rate (El_p_) of 3 baht per unit.

(5) $${\mathrm{Exp}}_{d}=\frac{1000\left(E\right)\left({\mathrm{El}}_{p}\right)}{m_{pi}\left(MC_{wi}-MC_{wf}\right)}$$

In Equation (5), Exp_p_ represents the drying expenses (baht·ton^−1^·kg^−1^); E is electricity energy consumption (kWh); El_p_ is electricity rate (baht·kWh^−1^); MC_w_ with subscripts i and f is initial and final moisture content on a wet basis.

## 3. Results and Discussions

### 3.1. Analysis of Ducting Strategies for Optimized Air Distribution in Paddy Piles

**Figure 7:**
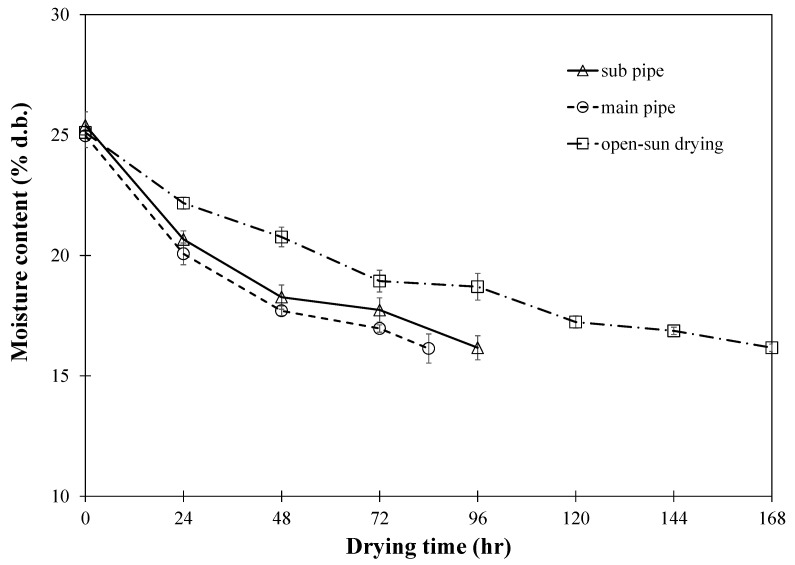
Drying kinetics affected by inlet air configuration.

In our study, we monitored changes in the moisture content of paddy rice within a traditional small-scale barn, comparing the efficacy of two distinct hot air-drying systems against the traditional sun drying method, as illustrated in Figure 7. Initial observations revealed a rapid decrease in moisture content, which slowed as the drying process progressed, reflecting the expected dynamics of moisture and heat transfer [14]. The goal was to reduce moisture from 25% (db) to an optimal level of approximately 16% (db). Our findings indicate that the hot air-drying methods significantly reduced drying time compared to sun drying. The superiority of moisture removal by hot air-drying, irrespective of the air distribution technique used, can be attributed to its effectiveness in lowering the vapor pressure around the grains, thus facilitating more efficient moisture extraction [15]. This process increases the grain temperature, enhancing the internal vapor pressure and promoting moisture migration from the interior to the surface [15]. Consistent airflow in hot air-drying further aids in uniform moisture removal, demonstrating its advantage over conventional sun drying methods in terms of speed and efficiency [16,17]. Despite the anticipatory nature of these outcomes, they validate the benefits of hot air-drying for small-scale agricultural applications, emphasizing its role in enhancing post-harvest management.

Considering the impact of hot air duct configurations, our findings showed that the method involving perforated sub-pipe distribution required approximately 96 h to achieve the target moisture content. In contrast, the system utilizing the main duct at the entrance proved to be more efficient, completing the drying process in roughly 84 h. The extended drying period associated with the perforated sub-air ducts can be attributed to a larger pressure drop within the drying system, as opposed to using the primary air pipe [3]. This is because the presence of the sub-air duct system causes the hot air to disseminate more slowly across the different layers of the rice stack, resulting in a more prolonged drying process. According to Razavizadeh et al. [3], this larger pressure drop, especially noted in configurations such as the F-duct due to increased turbulence and swirling, leads to slower hot air dissemination across the rice stack layers, thereby extending the drying duration. The H-duct configuration is recommended to minimize blind zones and ensure more uniform air distribution during continuous aeration processes, aligning with our objective to enhance drying efficiency and uniformity [3,18].

Hence, the in-barn paddy drying technique that utilized the perforated sub-pipe for hot air distribution was identified as the most suitable method for the subsequent research phases. This decision aligns with findings indicating that self-compaction plays a critical role in airflow resistance, which should be considered in the design and analysis of cooling aeration and low-temperature drying of in-store grain bulks [18]. Future research should focus on assessing airflow resistances under self-compaction for other grains, moisture content, bulk configurations, and airflow velocity ranges [18].

### 3.2. Temperature Distribution and Moisture Variation of the In-Barn Drying Paddy Process

In our study, we closely monitored the moisture content of paddy rice within a traditional small-scale barn, evaluating the performance of hot air supplied through perforated sub-air ducts at various horizontal and vertical locations in the pile. Our evaluations revealed uniform moisture levels across all vertical layers of the paddy pile (10, 20, and 30 cm), particularly noting that moisture content was consistent in the front, central, and rear sections of the barn. This uniformity in drying is critical, as consistent drying and preservation of rice grain quality have been underscored by studies such as those examining single-layer loading at low temperatures in silo-dryer-aerators [6].

Notably, the paddy located at a height of 30 cm displayed a marginally higher moisture content compared to those at 10 and 20 cm, especially in the front and rear sections of the storage area. We attribute these seasonal fluctuations and the resultant temperature gradients as significant factors influencing moisture movement within the grain, potentially leading to quality deterioration if not carefully managed [19]. Our findings support the notion that the specific inlet duct configuration and the resultant airflow pattern are paramount in effectively managing these temperature gradients and moisture movements.

Further analysis revealed consistent temperatures within the rice piles across all barn areas, irrespective of height. An interesting observation was that the inner temperature of the rice stack at 30 cm was slightly cooler than that at both 10 and 20 cm across all sections of the barn. This consistency in temperature, along with the observed gradients, plays a crucial role in the grain drying process, echoing the significance of temperature and humidity control in grain quality preservation, as indicated by studies emphasizing the use of dehumidifiers in grain storage [20].

The final moisture content data, presented in Table 1, showed relative uniformity at the bottom region of the pile across different horizontal positions within the barn. However, discrepancies in moisture content were more pronounced in the upper sections. This observation aligns with the understanding that mold activity remains subdued below certain moisture content thresholds, highlighting the importance of meticulous moisture and temperature management in stored grain to prevent quality loss [21]. Drying at specific temperatures, particularly higher temperatures, has been shown to significantly enhance seed longevity, assuming the seeds are harvested above a critical moisture content threshold [22].

The variations in moisture content observed at different layers during the drying process are corroborated by recent studies in grain drying and storage. These studies highlight the impact of single-layer loading at low temperatures for consistent drying and quality preservation of rice grains, pointing out differences in drying rates between lower and upper layers due to moisture diffusivity [6]. Additionally, the influence of seasonal atmospheric temperature fluctuations on moisture movement within the grain, leading to faster drying rates near air ducts and more uniform moisture content over time, further supports our findings [19].

Moreover, the critical role of controlling temperature and humidity to preserve grain quality during storage is underscored, particularly focusing on the effects of airflow through thermal space and grain parameters in a silo. These insights emphasize that proximity to airflow sources, such as air ducts, initially leads to significant variations in drying rates, which gradually even out to achieve uniform temperature and moisture levels throughout the storage [20].

By integrating these broader findings and insights from the literature, our study illuminates the intricate interplay between inlet duct configurations, airflow patterns, and storage conditions essential for achieving efficient temperature distribution and moisture content variation during the in-barn drying process of paddy rice. These insights not only corroborate the observations made in our study but also offer a comprehensive context for the observed patterns in temperature and moisture content variations, enhancing our understanding of efficient drying and uniform moisture content achievement in stored grains.

In our study’s comparison of the final moisture content, Khao Dok Mali 105 paddy rice, when dried by forced hot air supplied from the main air duct at the entrance of the storage facility, required 84 h to reach the desired moisture level. Upon assessing the final moisture content across various locations within the barn—specifically at the front, middle, and rear sections—it was observed that the front area exhibited a lower moisture content compared to the middle and rear sections, particularly at pile heights of 20 and 30 cm. This spatial variation in moisture content underscores the influence of air duct placement on drying efficiency. Moreover, when analyzing the moisture content at different pile heights—10, 20, and 30 cm—it became evident that paddy located at a height of 30 cm retained a higher final moisture content than those at lower heights across all sections of the barn. This observation highlights the challenges in achieving uniform drying throughout the paddy stack, suggesting that moisture removal is less effective at higher pile levels.

Furthermore, in considering the temperature distribution of the Khao Dok Mali 105 paddy rice, it was noted that the paddy piles at the front of the barn registered higher temperatures than those in the middle and rear, especially at a pile height of 30 cm. However, an assessment across varying pile heights revealed that the temperature at 30 cm was consistently lower than at both 10 and 20 cm, across the front, middle, and rear sections of the barn. This temperature gradient suggests that heat penetration is more efficient at lower levels of the paddy stack, impacting the drying process’s overall effectiveness.

The analysis of the data from Table 2 and Table 3 clearly showed that the perforated sub-pipe system enabled a more uniform distribution of hot air throughout the paddy stored in the small-scale barn, compared to the main pipe system. Despite this, the rice dried using the main pipe system also reached moisture levels deemed acceptable for storage. The introduction of hot air through the main air pipe at the barn’s entrance was found to be significantly more efficient in reducing moisture content. This method quickly lowered the paddy’s moisture from an initial 25% db to approximately 16% db, surpassing the performance of the perforated duct technique. The superior efficiency of the main air pipe method is likely attributable to the reduced pressure drop in the system, unlike the arrangement that included perforated sub-air ducts. As a result, our study pinpointed the main air pipe approach as the most effective strategy for dehydrating paddy within an in-barn setting.

This finding suggests that while both methods fall within acceptable storage moisture ranges, the main pipe system’s efficiency in moisture reduction offers a promising avenue for enhancing drying practices in small-scale agricultural settings. The implications of this efficiency gain are significant, suggesting that future research and practical applications might prioritize optimizing air flow and system design to maximize drying effectiveness and energy efficiency. By refining these systems, we can potentially improve post-harvest processing, reduce losses, and enhance the overall quality of stored grain.

### 3.3. Quality Attributes of Paddy Rice as Affected by Different Hot Air Ducting Strategies

Quality attributes of paddy rice dehydrated by using direct entrance ducting and perforated sub-pipe distribution were compared in terms of head rice yield, whiteness index, and sensorial properties to those dried by traditional open-air sun drying, as presented in Table 3.

Table 3 shows that the averaged head rice yield of paddy rice dried by all methods were not significantly different. This negligible variation can be attributed to the drying temperature set at 45 °C, which was relatively moderate. Consequently, the temperature disparity between the rice grain interior and exterior was minimal, resulting in a low moisture gradient and subsequently few instances of internal cracking within the grains. This led to a low percentage of broken rice, as reported by [6]. This study detailed the drying of paddy rice in a silo-dryer-aerator, noting a reduction in moisture content due to natural airflow over three months. Drying was conducted at temperatures above 25 °C, which favored grain quality by reducing stresses that often lead to cracks and breakage. Equilibrium moisture content reached 12% and 14% in the lower and upper layers, respectively. However, prolonged high moisture content in the upper layers caused heating of the grain mass, potentially impacting rice quality. Despite the variance in drying intensity between layers, the moderate drying temperature of 45 °C led to a negligible variation in the head rice yield, attributed to minimal temperature disparity and consequently low moisture gradient within the grains. This method effectively maintained a low percentage of broken rice, underscoring the importance of controlled temperature in preserving the quality and yield of paddy rice during drying. Wongpornchai et al. [23] investigated the impact of various drying methodologies and the duration of storage on the yield of head rice. It was observed that the yield of head rice was notably influenced by the drying techniques utilized. Particularly, when paddy was dried using hot air at 70 °C, the average yield of head rice was notably lower at 25.84%. Conversely, the yield was slightly improved when employing modified air at temperatures of 30 and 40 °C, and hot air at 40 and 50 °C, compared to traditional sun drying. The average yield for paddy dried using modified air (30 and 40 °C) and hot air (40 and 50 °C) was 56.48%, which is marginally higher than the 53.64% yield from sun drying. Furthermore, the study indicated minor variations in head rice yield across different drying treatments as the storage time progressed.

The study concludes that maintaining a moderate drying temperature, such as 45 °C, is pivotal in preserving the head rice yield during the drying process. This temperature regime minimizes the internal temperature disparity and moisture gradient within the grains, leading to fewer instances of internal cracking and a lower percentage of broken rice. While drying at excessively high temperatures, like 70 °C, significantly reduces the head rice yield, employing modified air at 30 to 40 °C or hot air at 40 to 50 °C slightly enhances the yield compared to traditional sun drying. Overall, the method and temperature of drying play a crucial role in maintaining the quality and yield of paddy rice, with moderate temperatures proving beneficial for minimizing grain damage and maximizing head rice yield.

When examining the average whiteness index of rice exposed to different drying techniques, the methods showed noticeable variations in whiteness. A marked difference in whiteness levels was statistically evident. Specifically, the rice dried through both hot air distribution strategies showed higher whiteness levels in contrast to those dried using the standard drying method, as highlighted in Table 3. Rice from the standard drying often exhibited a reduced whiteness, taking on a yellowish hue. This color shift could be linked to extended drying periods, possibly triggering enzymatic browning reactions and allowing the pigments from the bran and husks to seep into the grain.

When analyzing the effects of different drying methods on the whiteness index of rice, it is evident from the provided studies that the drying technique, temperature, and storage period significantly influence the color attributes of rice, which include lightness (L*), redness–greenness (a*), yellowness–blueness (b*), and whiteness index (WI). Hanisa et al. [24] highlighted that drying at higher temperatures can cause rice to darken due to enhanced browning reactions, reducing the whiteness index, mainly attributed to the browning reactions that occur at elevated temperatures during drying. Soponronnarit et al. [25] indicated that as drying temperature increases, the whiteness of rice decreases due to enhanced browning reactions. It further notes the impact of initial moisture content and tempering time on the whiteness, where a higher initial moisture content and longer tempering time resulted in a darker color of rice. The work conducted by Chitsuthipakorn and Thanapornpoonpong [26] delves into the changes in the whiteness index (WI) of white rice dried with different methods over a six-month storage period. It was observed that the whiteness index of white rice increased slightly during the storage, irrespective of the drying condition. This increase in whiteness index is potentially beneficial for rice trade negotiations. The subtle changes in color difference (ΔE*) throughout the storage suggest a certain degree of stability in the whiteness level. It was suggested that during drying and subsequent storage, starch granules in rice alter, forming a transparent layer that makes the rice grains appear shinier and reduces the enzymatic activity leading to browning.

In conclusion, while different drying methods and conditions affect the whiteness index of rice, carefully managing the drying temperature and moisture content can mitigate browning reactions and even improve whiteness over time. The increase in whiteness index during storage suggests that certain drying conditions can lead to a desirable shininess in rice, possibly due to changes in starch structure. This information is crucial for selecting drying methods that maintain or enhance the visual and marketable quality of rice.

Based on experimental findings from the two hot air distribution techniques in a traditional small-scale barn, the method using the main pipe system at the entrance proved quicker in reducing the moisture content from 25% db to the targeted 16% db compared to the perforated sub-air ducts. Yet, when assessing rice quality—particularly head rice yield and whiteness—both methods produced similar results. Given the research scope, the strategy deploying hot air from the entrance main duct was deemed most fitting for the traditional barn setup. Thus, rice dried using this method was further subjected to sensory quality evaluations and compared with rice dried via the conventional open-air sun method.

Table 3 reveals that the sensory attributes of the cooked rice dried using both hot air methods, such as aroma, whiteness, glossiness, cohesiveness, and tenderness, were on equivalence with rice dried traditionally in the sun. The prepared rice displayed a gentle aroma, bright and soft mature grains, and a mild cohesiveness. These features are consistent with the known characteristics of the KDM105 rice variety. Earlier studies have indicated that using lower drying temperatures (below 50 °C) helps preserve the volatile compound 2-acetyl-1-pyrroline, which gives the Khao Dawk Mali 105 rice its unique scent [27]. Additionally, the observed brightness in cooked rice is linked to the inherent whiteness of the milled variant [25]. As a result, this investigation determined that drying KDM 105 rice in a traditional barn yielded sensory qualities similar to the conventional open-air sun drying method. It underscored the feasibility of the in-barn paddy dehydration approach in preserving acceptable quality attributes.

### 3.4. Changes in Temperature and Moisture Content during Short-Term Storage

**Figure 8:**
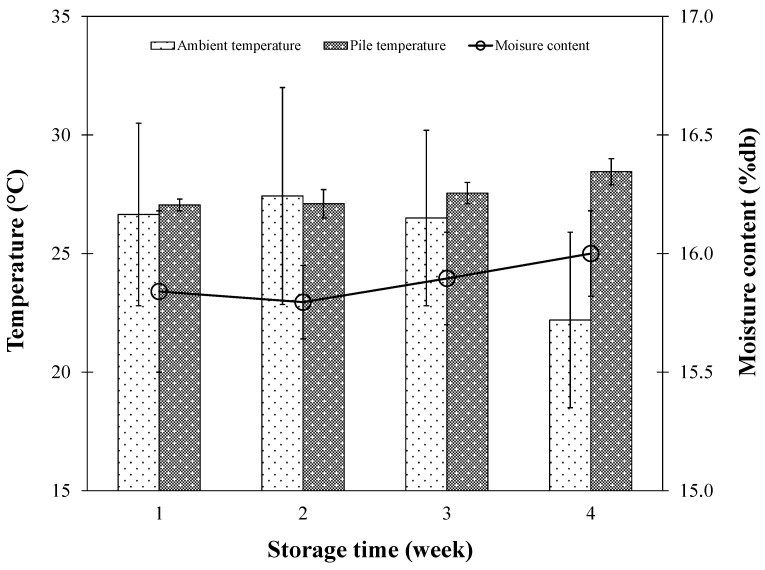
Change in temperature and moisture content as a function of storage time for one month.

In the initial month of storage within the traditional small-scale barn, the temperature within the rice pile showed an upward trend. Notably, during the final week, the temperature peaked at roughly 29 °C, noticeably surpassing the average ambient temperature, as depicted in Figure 8. This temperature elevation was likely due to the heat generated by the rice grains’ respiration, similar to the observations made in the study on freshly harvested rice during storage, which highlighted the slight increase in temperature due to respiration heat, affecting various properties of the rice, such as water absorption capacity and protein content [28].

A parallel observation was made by Phillips et al. [29] in their examination of a one-ton paddy pile, which had a moisture content ranging from 16.3% to 17.6% db and a height of 80 cm. This paddy was dried under natural conditions, where temperatures fluctuated between 36 and 42 °C. They observed that the central temperature of the paddy pile jumped from 36 °C to 46 °C within the first 30 storage days. This is consistent with the findings from another study that emphasized the need for thorough rapid drying and proper temperature control in bins to maintain quality, as temperatures typical of certain harvest seasons may degrade kernel quality in moist top layers of rice in a drying bin [30].

To counteract this heat buildup in paddy piles, it was crucial to introduce ambient air circulation to disperse this heat, particularly during the initial storage phase. The work of Millati et al. [28] further supports this by highlighting that drying paddy rice in a single thick layer at low temperatures preserved the physical quality, physicochemical constituents, and starch morphology of rice grains, suggesting that controlled temperature and moisture are key to maintaining rice quality during storage [6].

Nevertheless, this temperature surge during the early storage phase did not drastically influence the paddy moisture level, as indicated in Figure 8. Throughout the first storage month, the moisture content of the paddy grains remained relatively stable on a weekly basis, maintaining a moisture content of approximately 15.9% db. This corroborates the third document’s recommendation for prompt drying to 16% MC or less and storage at or below 27 °C for up to 10 weeks to prevent fungal growth and quality degradation due to post-harvest storage-induced yellowing [30].

### 3.5. Changes in Temperature and Moisture Content during Long-Term Storage under Occasional Forced-Air Ventilation

From the previously mentioned observations during the initial storage phase, the rise in the pile temperature had a negligible impact on moisture content. However, this temperature variation might have had implications beyond just moisture, potentially affecting the rice grain quality after extended storage. Notably, during the 6-month storage period covering the transition from winter to summer in Thailand, the relative humidity within the storage environment ranged from 52 to 78%. This fluctuation in humidity levels was carefully considered when selecting the optimal times for ventilation (11:00 a.m.–1:00 p.m.), during which the relative humidity was observed to be lower, ranging from 45 to 70%. This strategic choice in ventilation timing aimed to mitigate the impact of higher humidity levels on moisture content and grain quality, reflecting an understanding of the critical role that both temperature and humidity play in preserving rice integrity. In light of this, air ventilation was introduced to regulate the pile temperature and retain the paddy rice quality attributes. This approach resonates with findings from a study on drying paddy rice in a silo-dryer-aerator, which demonstrated the efficacy of low-temperature drying in preserving the physical and physicochemical quality of rice grains [6].

Figure 8 illustrates the relationship between pile temperature and storage duration in comparison with the monthly average ambient temperature. A noticeable increasing trend in pile temperature over time was evident, which might have been influenced by the seasonal transition from winter to summer. Despite this gradual increase, the monthly temperatures within the paddy stacks consistently stayed within the bounds of that month’s average ambient temperature, never surpassing 37 °C—a threshold supported by the study on the accumulation of respiration heat, highlighting the importance of maintaining specific temperature ranges to prevent nutritional degradation [28].

Research has indicated that during the static storage stage, grain respiration leads to initial heating at the bottom of the grain pile, forming a high-temperature core. In contrast, during the mechanical ventilation stage, as corroborated by a study using numerical simulations, cooling initiated from the air outlet spreads gradually to the entire silo, effectively lowering the grain pile temperature after significant ventilation [31]. This emphasizes the profound effect of controlled ventilation on temperature management and supports the strategic arrangement of air ducts for uniform temperature distribution. In addition, the study by Liu et al. [31] and the comprehensive storage study both emphasize that employing strategic air ventilation can effectively manage the temperature inside the paddy pile, aligning closer to the ambient temperature range and preventing excessive heat accumulation. This is critical, as even fully dried rice is susceptible to quality degradation under extreme temperature conditions. After ceasing the air circulation, a slight temperature increase in the paddy pile might be observed, possibly attributed to grain respiration. However, the overarching temperature management strategy remains effective, ensuring the preservation of rice quality throughout the storage period [30]. These observations are in line with practical outcomes, illustrating the critical role of removing excess heat through air ventilation in sustaining rice quality during storage.

**Figure 9:**
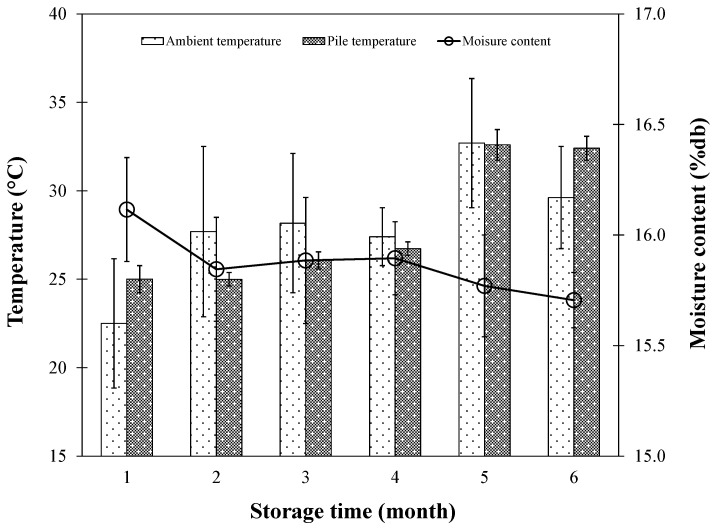
Change in temperature and moisture content as a function of storage time during long-term storage with occasional ambient air ventilation.

Moreover, in the present work, introducing ambient air ventilation once a month during a 6-month storage period resulted in only a minor change in the moisture content of the paddy rice grains, decreasing by approximately 0.4% db. Notably, the monthly moisture variations in the rice grains were not statistically significant, as depicted in Figure 9. This finding further supports the efficacy of the occasional ventilation strategy employed and substantiates its role in maintaining rice quality without significantly affecting the moisture content.

### 3.6. Quality Attributes of Paddy Rice during 6-Month Storage Associated with Air Ventilation

**Figure 10:**
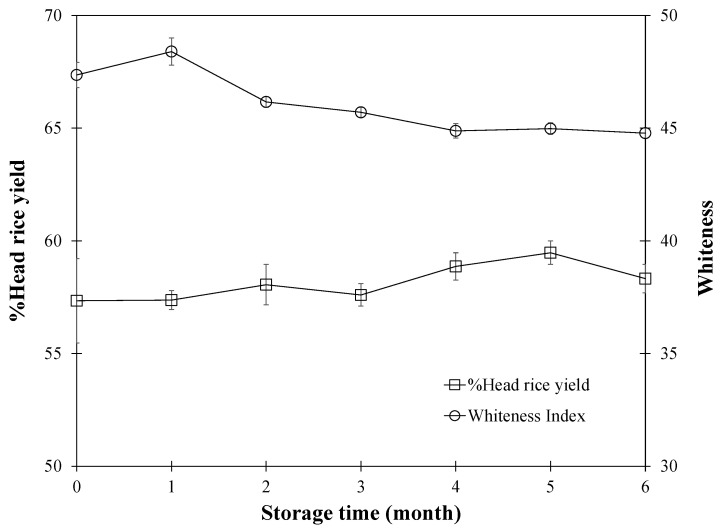
Change of % head rice yield and whiteness index as a function of storage time of 6 months.

Figure 10 illustrates the percentage of head rice yield (%HRY) throughout a six-month period, in conjunction with periodic ambient air ventilation. The data indicates that the %HRY remained largely constant, averaging 58.43%. This constancy is likely due to the regulated temperature and moisture levels within the storage environment, safeguarding the rice grains from milling damage. While this study focused on %HRY, whiteness, and moisture content as primary quality indicators due to their significant influence on consumer preferences and milling factory assessments, it is important to note that cooking properties and gelatinization characteristics were not examined in depth. The decision was based on the understanding that under our controlled storage conditions—ambient temperature and low storage temperature—significant changes in starch structure impacting these properties are unlikely. This approach aligns with industry standards for rice quality evaluation, emphasizing attributes that directly affect marketability and consumer acceptance. Future studies may explore these aspects under varying conditions to provide a more comprehensive understanding of rice quality dynamics [32]. This trend aligns with other studies indicating minimal fluctuations in head rice yield over storage durations [5,19,23]. Notably, there is evidence suggesting that %HRY may indeed increase during storage, as indicated by Soponronnarit et al. [25] and further supported by observations from Scariot et al. [4], who noted an enhanced whole grain yield and reduced broken grain percentage over time when white subgroup grains were dried at higher temperatures [4]. The underlying mechanism might involve the fusion of starch granules in the rice grains, reducing their susceptibility to breakage. Nevertheless, a decline in %HRY was observed after the initial three-month period, potentially due to the gradual moisture loss making the grains more brittle during milling.

With extended storage, the paddy rice is prone to discoloration, becoming more yellowish as a result of enzymatic reactions. The study’s examination of rice whiteness over six months revealed a decrease in whiteness of approximately 3.50% by the final month, yet maintaining a level above 37–38%, which falls within consumer-acceptable criteria [25]. A contributing factor to this reduction in whiteness could be the progressive rise in temperature within the paddy pile, a concern also highlighted by Moraes et al. [6], emphasizing the critical role of temperature regulation during drying and storage to prevent quality deterioration, such as grain breakage [6]. The empirical data indicates a notable rise in the paddy pile temperature during the latter months, likely accelerating enzymatic browning of the rice grains.

Moreover, the ambient conditions under which paddy is stored can significantly impact the rice’s whiteness, primarily due to the respiration process of the grains. This biological activity generates heat, raising the temperature inside the paddy pile. Consistent with findings from Prasantha et al. [33], rice stored in polypropylene and hermetic storage bags at a constant 30 °C showed a marked decrease in whiteness over nine months. The decline was from a range of 51.3–40.7% to 38.8–28.1% in polypropylene bags and to 36.1–23.6% in hermetic storage bags. Similarly, a study on Khao Dawk Mali 105 paddy rice stored at room temperature for six months reported a decrease in whiteness from 46% to 44% [25].

In summarizing, the investigation into paddy rice’s quality attributes during six-month storage with air ventilation concludes that %HRY predominantly exhibits stability, averaging at 58.43%. This steady state is largely credited to well-maintained temperature and moisture levels within the storage environment, which play a crucial role in preventing significant damage to the rice grains during milling. Nevertheless, the research points to the possibility of %HRY experiencing initial increases during storage, which eventually taper off, reflecting the intricate balance between storage duration and grain quality. Additionally, the study notes a gradual decrease in rice whiteness, particularly as the storage period concludes, primarily due to enzymatic browning and temperature elevation within the paddy pile. These insights underline the significance of optimal storage conditions for preserving paddy rice quality over time and contribute valuable knowledge to understanding the effects of ambient air ventilation on rice quality during storage. Such insights are instrumental in refining post-harvest handling and storage strategies.

### 3.7. Techno-Economic Results

An analysis of electric energy consumption for drying 1000 kg of paddy with an initial moisture content of 25% db reduced to roughly 16% db was conducted within the traditional small-scale barn using hot air distributed by two ducting systems. This analysis, as shown in Table 4, revealed that the paddy rice sample consumed less energy when dried using the main air duct method, registering an approximate specific energy consumption value of 1.87–1.94 MJ·kg^−1^. In contrast, using perforated sub-ducts resulted in a higher energy consumption of about 2.23–2.25 MJ·kg^−1^. This disparity can be attributed to the reduced drying time required by the main duct method, leading to decreased overall energy usage [34]. However, previous studies have shown that industrial-scale paddy storage facilities demand a specific energy consumption of roughly 2.88 MJ·kg^−1^ when utilizing forced ambient air to decrease paddy moisture from an initial 22% db (with a paddy stack height of 1.6 m) to approximately 16% db [35]. Furthermore, a study from a Malaysian rice mill employing an inclined bed dryer at approximately 42 °C found that drying 15 tons of paddy (with a stack height of 1 m) from an initial moisture content of 30% db to around 13.6% db necessitated about 5 MJ per kg of evaporated water [36].

During our six-month assessment of the traditional small-scale barn used for paddy storage, we monitored the electrical energy usage. Each two-hour session of introducing ambient air into the paddy stack consumed approximately 0.70 kWh per 1000 kg of paddy. This translated to an expenditure of 2.10 baht each time. Over the six-month period, the total electricity cost accumulated to 12.60 baht per ton of paddy stored.

## 4. Conclusions

This study explores the efficacy of hot air-drying and ambient air ventilation in enhancing paddy rice preservation within small barns, underlining the importance of controlled environmental conditions for rice quality. Notably, the head rice yield (HRY) showcased a stable average of 58.43% over six months, revealing the drying and ventilation methods’ capability of maintaining quality, despite observed fluctuations indicating the complex impact of storage conditions on grain quality. The research also identified rice discoloration towards the end of storage, suggesting the need for optimized conditions to prevent quality loss. Future research is encouraged to refine these processes using advanced technologies, aiming to bolster post-harvest handling and rice production sustainability. This contribution offers profound insights into improving agricultural outcomes through strategic drying and ventilation, promising enhanced grain quality and economic benefits.

## 5. Future Works

For future research, integrating advanced drying technologies like ultrasonic wave-assisted drying, tailored for small-scale barns and suitable for local farmers, is envisaged. This approach aligns with the innovative methodology presented in Dibagar et al. [37]. Emphasizing the importance of making these technologies accessible and practical, the application of numerical methods will serve as a critical tool for designing and optimizing the drying process. This strategy will ensure that advancements are both scientifically grounded and practically applicable, offering sustainable solutions for improving grain preservation.

## Figures and Tables

**Table 1:** Variation of final moisture content of paddy under different dehydration methods.

Location	Depth (cm)	Measuring Points	Moisture Content (% d.b.)	
			Sub Pipe	Main Pipe
Front	10	A1, A2, A3	15.50 ± 0.36 ^cNS^	15.40 ± 0.44 ^dNS^
	20	B1, B2, B3	16.00 ± 0.26 ^bcNS^	15.50 ± 0.40 ^dNS^
	30	C1, C2, C3	16.67 ± 0.25 ^abNS^	16.37 ± 0.38 ^bcNS^
Center	10	A4, A5, A6	15.90 ± 0.44 ^bcNS^	16.23 ± 0.32 ^bcNS^
	20	B4, B5, B6	16.03 ± 0.25 ^bcB^	16.77 ± 0.45 ^abA^
	30	C4, C5, C6	16.80 ± 0.30 ^abB^	17.37 ± 0.21 ^aA^
Back	10	A7, A8, A9	16.00 ± 0.70 ^bcNS^	16.00 ± 0.26 ^cdNS^
	20	B7, B8, B9	16.57 ± 0.70 ^abNS^	16.50 ± 0.46 ^bcNS^
	30	C7, C8, C9	17.40 ± 0.60 ^a^	17.20 ± 0.26 ^a^

Different small letters represent a significant difference between mean values in the same column (*p* < 0.05), while capital letters denote significant difference (*p* < 0.05) in the same row. NS stands for ‘not significant’.

**Table 2:** Temperature distribution in a paddy pile under different dehydration methods.

Location	Depth (cm)	Measuring Points	Moisture Content (% d.b.)	
			Sub Pipe	Main Pipe
Front	10	A1, A2, A3	32.17 ± 0.32 ^aNS^	32.70 ± 0.36 ^aNS^
	20	B1, B2, B3	31.00 ± 0.90 ^abcB^	32.43 ± 0.51 ^abA^
	30	C1, C2, C3	31.00 ± 0.92 ^abcNS^	31.13 ± 0.81 ^cNS^
Center	10	A4, A5, A6	31.67 ± 1.02 ^abNS^	31.40 ± 0.36 ^cNS^
	20	B4, B5, B6	30.73 ± 0.25 ^bcB^	31.50 ± 0.30 ^bcA^
	30	C4, C5, C6	30.10 ± 0.20 ^cNS^	29.87 ± 0.85 ^dNS^
Back	10	A7, A8, A9	31.37 ± 0.23 ^abNS^	31.83 ± 0.74 ^abcNS^
	20	B7, B8, B9	30.13 ± 0.75 ^cB^	31.63 ± 0.21 ^bcA^
	30	C7, C8, C9	29.80 ± 0.44 ^cNS^	29.57 ± 0.31 ^dNS^

Different small letters represent a significant difference between mean values in the same column (*p* < 0.05), while capital letters denote significant difference (*p* < 0.05) in the same row. NS stands for ‘not significant’.

**Table 3:** Quality attributes of paddy as affected by dehydration methods.

Quality Attributes	Dehydration Method		
	Sub Pipe	Main Pipe	Sun Drying
%Head rice yield ^ns^	56.03 ± 0.58	57.35 ± 1.91	55.49 ± 0.70
Whiteness	46.77 ± 0.31 ^a^	47.33 ± 0.85 ^a^	45.27 ± 0.45 ^b^
Sensory attributes of cooked rice			
fragrance ^ns^	3.68 ± 0.74	3.73 ± 1.01	3.45 ± 0.82
whiteness ^ns^	7.14 ± 0.52	7.36 ± 0.50	7.27 ± 0.47
glossiness ^ns^	7.25 ± 0.40	7.55 ± 0.52	7.09 ± 0.70
cohesiveness ^ns^	7.44 ± 0.65	7.36 ± 0.50	7.00 ± 0.00
tenderness ^ns^	7.18 ± 0.90	7.27 ± 0.47	7.00 ± 0.00

Different small letters represent a significant difference between mean values in the same row (*p* < 0.05), and ns stands for ‘not significant’.

**Table 4:** Efficiency of energy consumption in paddy dehydration under different techniques.

Drying Technique	MC_i_ (% db)	MC_f_ (% db)	M_evap_ (kg)	DT (h)	W_evap_ (kg·h^−1^)	E (MJ)	SEC (MJ·kg^−1^)	E-Bill (Baht)
Perforated sub pipe	25.17 ± 0.12 ^a^	16.32 ± 0.68 ^a^	75.10 ± 5.52 ^a^	96	0.78 ± 0.06 ^a^	167.04 ± 9.27 ^a^	2.23 ± 0.16 ^a^	1.86 ± 0.13 ^a^
Main pipe	25.37 ± 0.12 ^a^	16.37 ± 0.72 ^a^	75.87 ± 6.90 ^a^	84	0.90 ± 0.08 ^a^	146.16 ± 5.46 ^b^	1.94 ± 0.17 ^a^	1.61 ± 0.14 ^a^
Open-air sun drying	25.47 ± 0.21 ^a^	16.43 ± 0.38 ^a^	72.14 ± 2.40 ^a^	168	0.43 ± 0.01 ^b^	0 ^c^	0 ^b^	0 ^b^

MC_i_ and MC_f_ stand for moisture content at initial and final time, respectively; M_evap_—mass of evaporated water; DT—drying time; W_evap_—evaporation rate; E—total electric energy consumption; SEC—specific energy consumption; E-Bill—electricity bill. Different superscripts in the same column represent a significant difference at *p* < 0.05, while ns denotes ‘non-significance’.

## Data Availability

The original contributions presented in the study are included in the article, further inquiries can be directed to the corresponding author.
